# Diagnostic Lessons from a Complex Case of Postintestinal Transplantation Enteropathy

**DOI:** 10.1155/2017/2498423

**Published:** 2017-08-06

**Authors:** Cian Wade, Philip Allan, Elena Collantes, Srikanth R. Reddy, Peter J. Friend, Georgios Vrakas

**Affiliations:** ^1^University of Oxford Medical Sciences Division, Medical Sciences Office, JR Hospital, Headley Way, Oxford OX3 9DU, UK; ^2^Oxford University Hospitals NHS Foundation Trust, Oxford Transplant Centre, Churchill Hospital, Old Road, Oxford OX3 9DU, UK; ^3^Oxford University Hospitals NHS Foundation Trust, Translational Gastroenterology Unit, John Radcliffe Hospital, Headley Way, Oxford, Oxfordshire OX3 9DU, UK; ^4^Department of Cellular Pathology, John Radcliffe Hospital, Headley Way, Headington, Oxford OX3 9DU, UK

## Abstract

Recent advances in the field of intestinal transplantation have been mitigated by the incidence of allograft rejection. In such events, early identification and appropriate timing of antirejection therapy are crucial in retaining graft function. We present the case of a patient who suffered severe postintestinal transplantation allograft enteropathy, primarily characterized by extensive mucosal ulcerations, and was refractory to all conventional therapy. This progressed as chronic rejection; however crucially this was not definitively diagnosed until allograft function had irreversibly diminished. We argue that the difficulties encountered in this case can be attributed to the inability of our current array of investigative studies and diagnostic guidelines to provide adequate clinical guidance. This case illustrates the importance of developing reliable and specific markers for guiding the diagnosis of rejection and the use of antirejection therapeutics in this rapidly evolving field of transplant surgery.

## 1. Introduction

Recent surgical advances in the technical feasibility of intestinal transplantation (ITx) and improved posttransplantation immunosuppression mean that this is an increasingly explored therapeutic option for patients in intestinal failure [1]. Recent data report that 1-year intestinal graft survival is approximately 75%, whilst 10-year graft survival is 32% [2]. Whilst demonstrating that short-term outcomes are reasonable, medium to long-term patient morbidity is still significant. One major cause of graft failure is chronic rejection, with an estimated overall 8% incidence [3].

The diagnosis of intestinal allograft rejection involves a complex and ambiguously defined clinical assessment. Particular emphasis is placed upon histological studies of allograft biopsies, as well as an endoscopic and clinical evaluation of the patient [4–6]. Adjunctive investigations include radiological studies, serum biomarkers, and simple proxies of rejection, such as abdominal wall transplants [7, 8].  Table 1 lists the commonly used diagnostic features. Most, if not all, of these diagnostic features suffer from not being very specific for identifying a rejection event and are often found in several other pathological processes that lead to postintestinal transplantation enteropathy. The relative sparsity of high quality data identifying reliable indicators of rejection likely results from the historically relatively small numbers of intestinal transplantations performed compared to other solid organ transplantations [7, 9]. Given the importance of early interventions in antirejection therapeutics [10], as well as the dangers of unnecessary commencement of immunosuppressive drugs, there is a clear need for better evidence based guidelines that more reliably identify ITx rejection events.

Here we present the case of a 41-year-old gentleman who developed a nonspecific postintestinal transplantation enteropathy resulting in progressive loss of allograft function. This complex case illustrates potential difficulties encountered in diagnosing rejection events in patients who have received an intestinal allograft. We hope this case will encourage future work aimed at collecting prospective data contributing towards the development of reliable, noninvasive biomarkers of early rejection events.

## 2. Case Report

This report focuses on a 41-year-old Caucasian male with a background of severe inflammatory bowel disease (IBD). The patient received a small bowel, proximal colon, and abdominal wall transplantation at our unit in March 2014 after developing intestinal failure associated liver disease after a prolonged period of total parenteral nutrition.

The ITx functioned well for over a year with the patient regaining nutritional independence from the parenteral feed. His immunosuppressive regime was altered in April 2015 due to renal dysfunction with a switch from tacrolimus to sirolimus based immunosuppression. In September 2015, he presented to our centre with fevers, night sweats, and general malaise. A histopathological report of allograft biopsies taken upon initial admission demonstrated severely active acute ileitis and colitis, most in keeping with an infectious cause of his symptoms. However, immunostaining for EBV, EBER, CMV, HSV, and adenovirus and special stains for fungi were all negative. Other pertinent negatives from these initial studies included no excess of crypt apoptotic bodies, no blood vessel thrombi, and no evidence of dysplasia or malignancy. C4d staining and HLA-B37 Class I but not Class II antibodies were detected at a low level. A PET-FDG scan revealed pericolic inflammatory changes along the course of the transplanted large bowel with diffuse FDG uptake. These investigative findings provided contradictory accounts of the pathoaetiology of the patient's presentation. Favoured differentials at this stage included an infectious cause, a recurrence of IBD, or sirolimus induced ulceration.

Stoma output progressively increased and he became increasingly clinically malnourished as the degree of intestinal failure progressed. In November 2015, histopathological studies demonstrated patchy, ulcerated epithelium with some regenerative changes. Still no changes were seen in the superficial blood vessels visualised in these biopsy samples, decreasing the likelihood of a rejection episode and favouring the notion of a recurrence of IBD.

The extensive investigations employed up until this point had not successfully generated a single clear hypothesis as to the aetiology of this gentleman's presentation and were inconsistent with his response to empirical therapies. Despite receiving increasing steroid doses, the patient continued to deteriorate with intermittent septic episodes.

**Figure 1:**
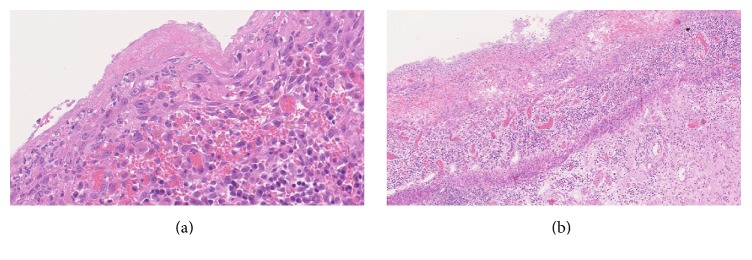
Histological sections of the transplanted bowel taken in April 2016 prior to complete explantation. (a) shows ulceration with a fibrinous exudate and granulation tissue formation consistent with severe colitis. (b) shows ulceration to the level of the muscularis propria as well as abundant ulcer slough consistent with our assertion that the regenerative state of the bowel could not be assessed adequately by histology.

**Figure 2:**
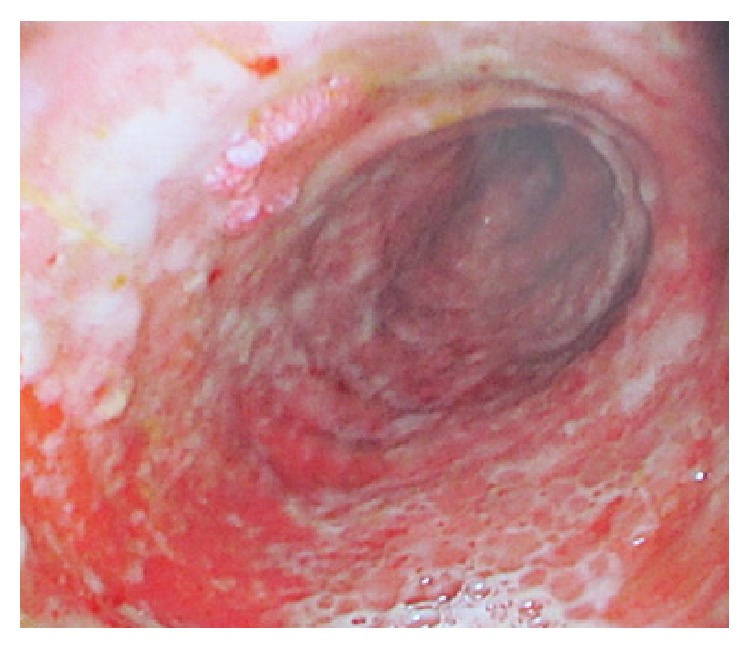
Macroscopic appearance of the bowel on endoscopy prior to right hemicolectomy in February 2016. This image demonstrates a severely circumferentially ulcerated transplant ileum consistent with severe rejection.

In early December 2015, the patient developed an erythematous rash localised to his abdominal wall allograft. Histological analysis of an abdominal wall sample revealed Grade 3 rejection (Banff classification) with apoptotic keratinocytes in the basal cell layer. Subsequent ITx biopsies revealed increased numbers of apoptotic bodies in the crypts in association with severe mucosal ulceration and granulation tissue, now suggestive of combined acute rejection of both abdominal wall and intestinal graft (Figures 1(a) and 1(b)). Plasma citrulline levels demonstrated a progressive downward trend with levels consistently below 10 micromol/L from this period in December 2015 onwards. Over the same time course, faecal calprotectin levels also progressively increased. However, despite continued steroid and antithymocyte globulin therapy, he suffered ongoing intestinal inflammation and based on the macroscopic appearances of his transplanted bowel (Figure 2()), a right transplant hemicolectomy was performed in February 2016.

Based on previous positive experience [12], we used mesenchymal stromal cells (MSC) as a last resort treatment to salvage graft function. Two empiric cycles of intravenously administered autologous mesenchymal stromal cell therapy (two doses of 2 million cells/kg one week apart) were commenced in March 2016. However, no clinical improvement was observed. The decision was therefore taken to proceed with total explantation of the remaining bowel and abdominal wall transplant in April 2016. Histological studies of the explanted allograft revealed features of mucosal infarction and necrosis along with perivascular inflammatory changes suggestive of chronic rejection. He has since been discharged home on total parenteral nutrition and awaits a second intestinal transplant.

## 3. Discussion

We present the case of a gentleman in whom exhaustive investigations of nonspecific posttransplantation enteropathy over a number of months failed to definitively identify the underlying pathological processes. Our patient had a complex and evolving clinical and histological pattern of disease and amongst the considered differential diagnoses were infection, sirolimus induced ulceration, recurrent IBD, posttransplant lymphoproliferative disease, or a form of acute or chronic rejection. Retrospectively, the temporal relationship between his switch from tacrolimus to sirolimus based immunosuppression leads us to contest that his initial presentation was most likely due to the previously described phenomenon of sirolimus induced bowel ulceration [13]. This ulceration increased the circulating allograft antigenic load and led to the development of the ITx rejection event in December 2015. The delay in diagnosis of this rejection event by a number of months and the consequently inappropriate immunosuppressive regime eventually led to allograft failure. We suggest that this diagnostic delay can be largely attributed to the lack of specificity of current diagnostic features for rejection events. In order to provide adequate clinical guidance, it is imperative that our diagnostic guidance has a good sensitivity for the early detection of rejection as well as the ability to reliably differentiate between multiple possible underlying aetiologies. Clearly this distinction is of particular importance for certain pathological processes, such as infection and rejection events, given that the therapies employed for each are diametrically opposed.

A crucial diagnostic issue in this case was an overreliance upon the currently regarded gold-standard investigation of histopathological studies. We identified three key reasons for the difficulties encountered in making a histopathological diagnosis of rejection in this case: firstly, chronic rejection characteristically involves fibrosis and stenosis of the bowel vasculature, typically the submucosal or mesenteric arteries. However, endoscopic biopsies typically do not have sufficient depth to thoroughly assess this vasculature [14]. Secondly, ulceration of the mucosa (Figures 1() and 2()) meant that the regenerative state of the bowel could not be easily assessed. The proportion of cells with high nucleus: cytoplasm ratio is commonly used as a proxy for the efficacy of immunosuppressive treatments as it indicates that sufficient immunomodulation is being provided to allow endogenous regenerative mucosal growth [15]. Without this histological proxy, we were limited in our ability to monitor the success of our interventions and also to infer the ongoing pathological processes. Lastly, mucosal inflammation and ulceration were noted early in the series of examined biopsies (from September 2015 onwards). These features are key tenets of the consensus histological classification system developed for diagnosing small bowel rejection, along with features such as lymphocytosis and crypt apoptosis (Table 1). However, these are very nonspecific histological findings and are features that largely overlap with the other aforementioned potential pathoaetiologies, including recurrent IBD and viral infections [4, 5, 16].

Although histopathology is regarded as the gold-standard investigation, it is widely accepted that this must be integrated with other clinical findings in order for a diagnosis of rejection to be made. However, clinical and endoscopic diagnostic features of ITx rejection are also shared by several other possible causes of posttransplantation enteropathy [16]. Other biomarkers employed in this case also failed to convincingly point towards a diagnosis of rejection. Fluctuations in the levels of biomarkers such as faecal calprotectin and plasma citrulline implied underlying graft rejection, but their validity in reliably identifying underlying pathoaetiology is contested [17, 18]. Additionally, in the absence of clear microscopic and macroscopic evidence of rejection, the presence of Class I anti-HLA-B37 antibodies alone does not strongly point towards a diagnosis of rejection. C4d is also not validated in reliably indicating antibody mediated rejection of intestinal transplants [19]. Indeed, a relative lack of rigorous, prospective studies renders much of the evidence relating to biochemical markers of intestinal rejection unsuitable for strongly influencing day-to-day clinical decision making.

Another crucial issue in this case was that this diagnostic uncertainty led to a time delay in employing appropriate therapeutic measures, ultimately leading to the failure of our salvage attempts. Exciting, emerging technologies aimed at improving allograft function often rely upon the early identification of rejection. An example in this case was the administration of MSCs in March 2016. Case series studies and our own centre's experiences suggest that MSCs can improve outcomes in patients suffering from ITx dysfunction [12, 20]. However, the failure of the MSCs to improve this patient's clinical outcome is likely due to the cells being administered too late at a time when crypt endogenous stem cells had already been lost due to the prolonged rejection process [21, 22]. This specific example highlights the wider issue of current diagnostic criteria often leading to a delay in definitive diagnosis and therefore mitigating attempts at ameliorating rejection events and rescuing graft function.

Innovative metabolomic and proteomic work aimed at identifying novel, noninvasive biomarkers of rejection is an example of where progress is being made in regard to improving the specificity of diagnostic features [23, 24]. Candidates for more reliable markers include leukotriene E_4_, taurocholate, vitamins B_2_, B_5_, and B_6_, and microRNA expression profiles [23, 25]. Additionally, abdominal wall allografts may show histological evidence of rejection processes prior to rejection occurring in the intestinal allograft, thereby acting as a sentinel early warning sign of impending ITx rejection [8]. However, clearly further work is required to ensure that these prospective methods are validated and can reliably be used as corollary clinical tests when considering a diagnosis of ITx rejection.

## 4. Conclusions

This was a highly complex case of postintestinal transplantation enteropathy of unknown aetiology. The case highlights the need for the development of more comprehensive and specific criteria for identifying ongoing pathology in intestinal allografts. An overreliance upon evolving histopathological findings in conjunction with other nonspecific findings from the clinical, endoscopic, radiological, and biochemical investigations led to an ultimately critical delay in diagnosis for our patient. This delay likely resulted in the failure of his allograft and perhaps the failure of our antirejection therapeutics. With the increasing popularity and viability of intestinal transplantation, we hope that our case provides an impetus for the systematic collection of prospective data from postintestinal transplantation cohorts aimed at identifying reliable and preferably noninvasive biomarkers and predictors for rejection events. In this way, the long-term outcomes for these patients will significantly improve and this exciting and emerging area of transplant surgery will continue to grow.

## Figures and Tables

**Table 1:** Diagnostic features of acute cellular intestinal allograft rejection.

Histological [4, 5]	Endoscopic [11]	Clinical features [6]	Biochemical [7]
Increased apoptotic bodies in crypts^2^	Oedema, erythema	Abdominal pain	Increasing faecal calprotectin levels
Crypt epithelial injury	Villous blunting	Fever & vomiting	Decreasing serum citrulline levels
Distortion of villous and crypt architecture	Loss of mucosal vascular pattern and friability	Increased output from stoma	Presence of allospecific CD154þ T cells increases risk
Mucosal ulceration^1^	Mucosal ulceration^1^	Septic shock^1^	Presence of DSAs increases risk

^1^Occurring in severe acute cellular rejection. ^2^Requiring >6 per 10 crypts.

## Abbreviations

CMV: Cytomegalovirus
EBER: Epstein-Barr virus-encoded small RNAs
EBV: Epstein-Barr virus
HSV: Herpes simplex virus
IBD: Inflammatory bowel disease
ITx: Intestinal transplantation
MSCs: Mesenchymal stromal cells.
